# Not “just a VUS”

**DOI:** 10.1016/j.gimo.2026.104401

**Published:** 2026-04-29

**Authors:** Christina G. Tise, Sophia M. Adelson, Elizabeth B. Garrity, Devon E. Bonner

**Affiliations:** 1 Division of Medical Genetics, Department of Pediatrics, Stanford University, Stanford, CA; 2 Founder, Citizen Genetics

In the Spring of 2025, I cared for the second child of a couple who ultimately died of “presumed” TRMU deficiency at 8 weeks of life. I knew this family well because I had cared for the couple’s older child about a year-and-a-half prior in November 2023 upon presenting with acute onset lactic acidosis and cardiomyopathy that resulted in an anion gap of >70 and death in the neonatal intensive care unit. In evaluating their older child in Fall of 2023, trio rapid genome sequencing from laboratory A was ordered and demonstrated a homozygous variant of uncertain significance (VUS) in *TRMU* (NM_018006.4:c.652-6_652-2del, splicing) with biparental inheritance, consistent with the clinical presentation and laboratory findings seen in TRMU deficiency.^1^^,^^2^ At the time I discussed this case and variant with a renowned expert in TRMU deficiency, who stated “I think it’s probably more severe since some of the protein is completely lost, whereas the “milder” kids tend to have missense or 1-3 amino acids in-frame deleted on at least one allele.”

This case prompted the development of the Variant Reclassification Project,^3^ or Starfish Project,^4^ aimed at assessing the yield of successful variant reclassification requests for variants with strong clinical and/or laboratory evidence of pathogenicity. Clinicians were asked to nominate cases that they felt had the strongest clinical and/or laboratory evidence of a specific genetic disorder with ≥1 VUS in a gene associated with the disorder. The Starfish Team compiled supporting patient data into formal requests for variant reclassification and submitted them to the reporting clinical laboratories. Time points in this process were tracked, including time spent compiling and drafting the reclassification request (step 1) and time between request submission and the laboratory’s determination response (step 2). The average time to complete step 1 was 63 minutes (range 25-111), with requests submitted to 5 clinical genetic testing laboratories. On average, step 2 took 12.9 days (range 1.1-113.7). Only 5 of the 34 (14.7%) requests resulted in VUS reclassification to pathogenic or likely pathogenic.^3^

The request for reclassification of the above *TRMU* variant was included in the Starfish Project. Compiling the variant reclassification request took 78 minutes and was submitted to laboratory A on March 6, 2024. Laboratory A responded 6 days later with their decision to not upgrade the *TRMU* VUS due to insufficient evidence. Specifically stating that application of variant scoring criteria PM2, PP3, and PP4 alone did not provide enough support for reclassification without definitive proof that the splice variant results in loss-of-function, which then also prevented the application of PVS1 (see Supplemental Information for additional details).

Seventeen months later in April 2025, I found myself in the Cardiovascular intensive care unit at the bedside of this couple’s newborn child, sharing with them the same slides and handouts on TRMU deficiency that I had used during their older child’s hospitalization. This newborn child was presenting at the same age (8-weeks-old) with essentially identical clinical and laboratory features, including an anion gap that rose to >80 and then became incalculable. As expected, the same homozygous splice variant in *TRMU* was identified by rapid genome sequencing that resulted postmortem. Once again, the homozygous *TRMU* variant was classified as a VUS, but this time by laboratory B, which had been strategically chosen with the hope that laboratory B would classify the variant differently based on the family history and clinical features and/or presence of this variant in their database. The genetic testing reports and clinical records for both siblings were shared with laboratory A and laboratory B on April 8, 2025, with the request to reclassify this variant to likely pathogenic or pathogenic; this request was declined by both labs.

Laboratory A’s response (April 16, 2025): “After review, our genomic analysts and geneticists shared that although we are still internally suspicious that the TRMU c.652-6_652-2del variant may be pathogenic, we still formally classify it as a VUS.”

Laboratory B’s response (April 21, 2025): “Although we agree that this is a strong variant, we are unable to upgrade the classification from a VUS at this time. We would need to observe this variant segregating in another affected relative/in other affected families or need a functional study to confirm the effect of the splicing variant. At this time, in silico analysis is inconclusive as to whether the variant alters gene splicing.”

As a Medical, Biochemical, and Perinatal Geneticist practicing at a children’s hospital that serves a diverse patient population, including those underrepresented in genomic databases, I have seen and published on primary ancestry-related health care inequities and secondary reproductive inequities stemming from disease-causing variants that are classified as VUS.^5^, ^6^, ^7^ Although I appreciate that clinical laboratories do their best to classify variants based on available information and established guidelines, the experience of being the counseling clinician for this underrepresented family and explaining to them why their familial disease-causing variant is considered a “VUS” by 2 separate laboratories after the death of a second homozygous child was gut-wrenching.

The impact of accurately reclassifying a single disease-causing VUS can be monumental and life-changing because it can provide a family with a diagnosis and prognosis, access to timely reproductive counseling and testing options, and improve genetic testing for future patients harboring the same variant. The most illustrative case at our institution has been the realization and recognition of the *PEX6* missense founder variant (NM_000287.4:c.1409G > C[p.Gly470Ala]) among individuals of Mixtec ancestry that causes a severe, neonatal lethal form of Zellweger spectrum disorder.^8^ This variant was initially classified as a VUS by laboratory C in the first infant we diagnosed clinically and then again by laboratory A in the second infant we diagnosed only months later. The variant was eventually upgraded by both laboratories at some point between the first and the ninth infant diagnosed but only after several clinicians from multiple nearby institutions spent significant time and effort providing the laboratories with relevant clinical information.

The challenges presented by VUS for providers, patients, and clinical laboratories are well known in the field of Medical Genetics. In 2020, the National Human Genome Research Institute predicted that VUS would be obsolete by 2030. In January of 2024, Fowler and Rehm^9^ argued that “many, if not most, VUS in coding regions” would be resolved by the predicted date, describing a welcomed world where “with high-quality predictions and functional data available for a gene, along with information from previously sequenced individuals, the overwhelming majority of single-nucleotide variants could be classified to a level that would enable a clinician to act on genetic information in the care of a patient, as well as identify borderline VUSs for which an incremental amount of additional evidence could push variants over to likely pathogenic.” As discussed by Rehm et al,^10^ diagnostic laboratories are making efforts to support patients and providers while the Clinical Genome Resource (ClinGen) Sequence Variant Interpretation Working Group works to revise the variant interpretation guidelines. “To further aid in VUS reporting and provider follow-up…some laboratories use sub-tiers of VUS …providers have relayed that these distinctions are helpful to guide which variants to follow-up on and which to ignore.”^10^ However, until guidelines are published, reporting policies continue to vary between laboratories and even among assays at the same laboratory (eg, copy-number variant reporting by chromosomal microarray versus genome sequencing).

This commentary serves several purposes. First is the hope that a publication about this *TRMU c.652-6_652-2del* variant will be the tie breaker in upgrading this variant to pathogenic or at least raise awareness of this variant’s association with TRMU deficiency. Perhaps then the parents of these 2 children will finally be able to provide another health care provider a diagnostic report that is unambiguous regarding their reproductive care and counseling. A genetic testing report demonstrating that both parents are heterozygous for a pathogenic variant in *TRMU* would be particularly actionable for this couple because there is evidence to support the use of L-cysteine and N-acetylcysteine in utero for TRMU deficiency.^11^ Despite available therapy for this condition, there is currently no other way for these parents to obtain a genetic testing report clarifying their carrier status for this condition, as *TRMU* is not included on most carrier screening panels,^12^ and even if it were, VUS are not typically reported by most laboratories offering carrier screening.^5^ Furthermore, many clinicians, medical societies, and health care systems are reluctant to test pregnancies for variants classified as VUS,^13^ which significantly limits couples’ reproductive options and prevents the opportunity to initiate treatment prenatally and/or immediately after birth.^5^^,^^6^

Second is to ask that the American College of Medical Genetics and Genomics (ACMG) and Association for Molecular Pathology (AMP) address and clarify the statement “A variant of uncertain significance (VUS) should not be used in clinical decision-making,” in the next version of Standards and Guidelines for the Interpretation of Sequence Variants.^13^ As written, the current guidance may be interpreted to suggest that the results of genetic testing in over half of the patients cared for at our institution should be disregarded. Although it is often the case in the field of Cancer Genetics that a VUS is later downgraded to “likely benign” or “benign,”^14^ this is not necessarily true of broad phenotype-driven diagnostic genetic testing among affected patients, particularly those of underrepresented ancestries. The 2015 ACMG guidelines also state that “Efforts to resolve the classification of the variant to “pathogenic” or “benign” should be undertaken” but do not to specify the party responsible for such efforts.

Third is to commend and support efforts to improve upon the current variant classification criteria used for clinical genetic testing and reporting^15^^,^^16^ and to request that some form of quantitative or qualitative value be given to the clinical impression of the Medical and Biochemical Geneticists, who are experts, often painfully so, in the specific and discriminating phenotypic features of these ultrarare conditions. For the *TRMU* c.652-6_652-2del familial variant presented, current guidelines only allowed for the application of 1 supporting criteria (PP4) despite overwhelmingly strong clinical and biochemical evidence. However, Biesecker et al^16^ have more recently proposed that for disorders with high diagnostic yield due to phenotypic specificity, such as TRMU deficiency, the PP4 evidence criterion can be applied significantly higher than supporting. Furthermore, this heuristic acknowledges that although the specific phenotype criterion (PP4) is inseparably coupled to the cosegregation criterion (PP1 and BS4), if PP4 has not been maxed out, PP1 segregation data can still be additive. This suggests that the presence of this homozygous variant in 2 similarly affected full siblings born to heterozygote parents should increase the strength of the combined PP1/PP4 criterion.^16^

The Starfish Project is actively working on adding deidentified, disease-specific clinical data to publicly available variant databases with fields available for this purpose, such as ClinVar (https://www.ncbi.nlm.nih.gov/clinvar), Franklin (https://franklin.genoox.com), and Varsome.^17^ ClinVar exists to standardize variant classification, and clinical laboratories are encouraged to contribute to variant databases, but guidelines and incentives would support timely and complete reporting. As explained by Deverka et al,^18^ “Laboratories cite numerous hurdles to public data sharing, such as a lack of staff and time, leading to unrecouped costs and other financial burdens.” Payors may have a role in encouraging improved data sharing practices by providing financial incentives to both laboratories and physicians for submission of evidence to publicly available data sets to aid in accurate variant classification, ultimately for the benefit of all patients.

The fourth purpose is a call to action for increased data sharing and an improved system for genetic testing companies to collaborate with clinicians to upgrade or downgrade variants for the next patient or family. Currently, clinicians provide genetic testing laboratories with relevant clinical data and in vivo functional studies (ie, clinical laboratory results) to aid in variant interpretation. The cost, of which, is often shouldered onto the clinician as nonreimbursable time and effort,^3^ the patient or their insurance when additional clinical testing is ordered (eg, broader testing, methylation studies, or RNA sequencing), or research funding for projects that support functional studies, such as the GREGoR Consortium.^19^^,^^20^ Ultimately, this results in each individual testing laboratory becoming the steward of this rich phenotype data, which often remain siloed and unshared. The ClinGen Sequence Variant Interpretation Working Group has provided recommendations for using the current guidelines to incorporate evidence for splice variants suggested to have a predicted or observed impact on splicing^15^; however, it is unlikely that application of this recommendation would have impacted the classification of the *TRMU* c.652-6_652-2del variant by either laboratory A or B based on the predicted impact on splicing shared with us by laboratory A: “It weakens the canonical splice site, but the deletion still results in an AG at the junction. Given this, we cannot apply PVS1, as it is not clear if this really impacts splicing or not. In addition, if this variant resulted in exons skipping, it would be an in-frame deletion of 18 amino acids and wouldn't appear to create a frameshift,” (see Supplemental Communication with Laboratory A & B for additional details). But even if one of our *TRMU* siblings had been enrolled in a functional research study before their death that was able to confirm aberrant splicing, as recommended by laboratory B, it remains unclear whether this information would be considered because our experiences suggest different genetic testing laboratories have different standards when it comes to using research data as clinical evidence in variant vetting. All parties involved would benefit tremendously from guidance on this because the time from variant discovery to peer-reviewed publication for rare diseases can take decades, delaying meaningful diagnosis of patients and families in the meantime.

Finally, this commentary serves to demonstrate that a VUS is not always “just a VUS” and highlight the dark gaps in health care, large academic hospitals included, that fail patients with rare genetic disease, especially those underrepresented in biomedical research. I hope through collaboration between clinicians, clinical laboratories, and patient advocacy groups we can find a more effective, equitable, and efficient path forward. Reassuringly, the paving of this path is actively underway, with input from several key organizations and thought leaders, with recommendations for quantitative/qualitative weighting of several variant scoring criteria (PP1, PP4, PP3/BP4, PS3/BS3)^15^^,^^16^ already being applied as best practice recommendations.^21^ Significant changes are forthcoming in the highly anticipated ACMG/AMP/CAP/ClinGen v4.0 Standard for Sequence Variant Classification, including the subclassification of VUS by likelihood of pathogenicity. The hope is that this VUS subclassification scheme offers the field the ability to disregard only VUS that are very unlikely to be relevant to a patient’s health. Research piloting VUS subclassification schema by Bennet et al^22^ has been encouraging, finding that, “variants in the lowest subclass of VUS were never reclassified as likely pathogenic (LP) or pathogenic (P).” However, until genomic databases are better diversified, novel variants that have never been observed, nor functionally characterized, will continue to surface in mass (appropriately so) as long-overdue genomic diversification efforts are implemented. Ultimately, a critical skill in caring and treating patients and families with rare and ultrarare disease is the ability to counsel and make recommendations in a thoughtful, transparent, and honest manner, with the willingness to acknowledge what is and is not certain. Only then can we engage in a shared decision-making model that simultaneously acknowledges the dynamism of our beloved field while also respecting the needs and autonomy of patients.

## Conflict of Interest

The authors declare no conflicts of interest.
