# Supplementary material for: Not “just a VUS”
Source: Genet Med Open. 2026 Apr 29;4:104401. doi: 10.1016/j.gimo.2026.104401 (PMC13240777; doi:10.1016/j.gimo.2026.104401)
Supplement: Supplemental Information [file mmc1.pdf]

Supplementary Information:

Summary of Communication History with Laboratory A Regarding Older Sibling

March 6, 2024: *TRMU* (NM_018006.4:c.652-6_652-2del, splicing) variant reclassification request to Laboratory A

- Patient harbors a homozygous VUS in the *TRMU* gene c.652-6_652-2del (splicing variant) with a disease presentation consistent with TRMU deficiency.
- We are requesting review of this genotype-phenotype correlation and for this variant to be reclassified as pathogenic or likely-pathogenic.
- Clinical Presentation
  - Presented at 20 hours-of-life with lactic acidosis, cardiomyopathy, elevated ammonia, and hypotonia.
  - Patient is now deceased, passing away at 8-weeks of age from respiratory failure attributable to their TRMU deficiency.
- This diagnosis is further supported by the following evidence:
  - Lactate elevated (ranging 16-17) with worsening heart function.
  - Patients with different variants in *TRMU* have presented with findings of TRMU deficiency which consist of mitochondrial dysfunction with liver failure and severe persistent lactic acidosis, hypoglycemia, hyperammonemia.
  - This variant is homozygous and TRMU deficiency is inherited in an autosomal recessive manner.
  - Email correspondence with TRMU deficiency expert : "I think it's pathogenic and I think it's probably more severe since some of the protein is completely lost, whereas the 'milder' kids tend to have missense or 1-3 amino acids in-frame deleted on at least one allele, which to me I think makes it more likely to explain 'mito dysfunction' more globally and the HCM."
  - Clinical laboratory measurements
    - Lactate - 19.44 mmol/L (ref <2)
    - Sodium, Ser/Plas - 180 mmol/L (ref 135-145)
    - Potassium, Ser/Plas - 6.1 mmol/L (ref 3.5-6.0)

- Chloride, Ser/Plas - 80 mmol/L (ref 98-107)
- Anion gap - 65 mmol/L (ref 5-15)
- Ammonia - 180 mmol/L (ref < 45)

o Pediatric echocardiogram: Some systolic anterior motion of the mitral valve apparatus in association with significant left ventricular hypertrophy without a significant left ventricular outflow tract gradient. Normal-sized right ventricle with moderate hypertrophy and mild-to-moderately depressed systolic function. Normal-sized left ventricle with severe concentric (increased mass-to-volume ratio) hypertrophy and normal systolic function. Moderate circumferential pericardial effusion, worse compared to the previous study.

o Cardiology Consult Note: "...clinical status improved after NPO status, high GIR and initiation of N-acetylcysteine and L-cysteine"

o Abdomen Ultrasound: Liver diffusely echogenic and enlarged.

- Attachments to email
  - o Neonatal Intensive Care Unit (NICU) admission note (11/20/23)
  - o Laboratory A genetic testing results (reported 12/2/2023)
  - o Medical Genetics follow-up consult note (11/27/23, 1/2/24)
  - o Abdominal ultrasound report (11/23/23)
  - o Cardiology consult follow-up note (1/10/2024)
  - o Graph of lactic acid level

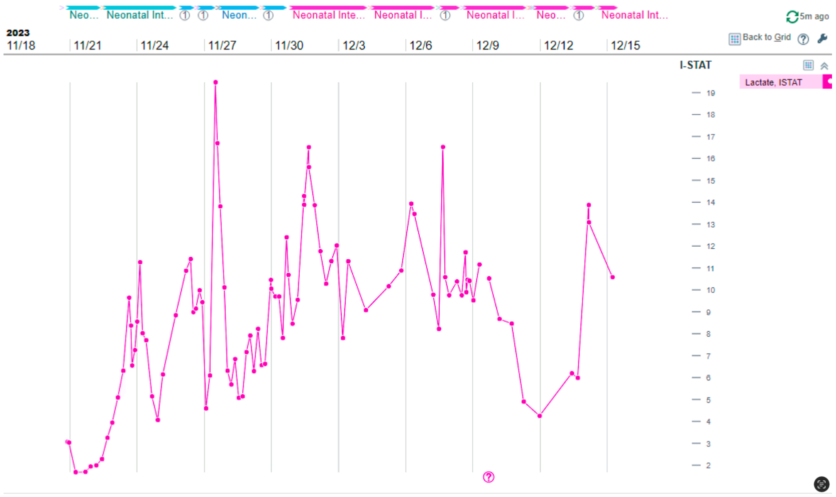

March 13, 2024: Summary of Laboratory A's response to Clinical Team

We applied the following ACMG criteria:

- PM2: Absent from controls or at extremely low frequency if recessive
- PP3: Multiple lines of computational evidence support a deleterious effect on the gene or gene product
- PP4 :Patient's phenotype or family history is highly specific for a disease with a single genetic etiology

Even with PP4 accounted for using the information you shared, the variant does not meet the threshold to be classified as likely pathogenic or pathogenic. The main reason we are unable to reclassify this variant is because it is not a clear LOF splice variant. It weakens the canonical splice site, but the deletion still results in an AG at the junction. Given this, we cannot apply PVS1, as it is not clear if this really impacts splicing or not. In addition, if this variant resulted in exons skipping, it would be an in-frame deletion of 18 amino acids and wouldn't appear to create a frameshift.

April 8, 2025: Second *TRMU* (NM_018006.4:c.652-6_652-2del, splicing) variant reclassification request to Laboratory A

Hi, Attaching the prelim result from [Laboratory B] on the full sibling of this proband. I am requesting an upgrade of this VUS in the gene *TRMU*. I have attached the clinical notes for our patient (who passed away this morning) and his sister who passed away last year with exactly the same clinical presentation, down to the week of life. Please reach out with questions or if you need additional data, as I have zero doubt this variant is pathogenic.

Attachments to email

- Laboratory B genetic testing results from younger sibling (reported 4/8/2025)
- Medical Genetics consult follow-up note from younger sibling (4/7/25)
- Medical Genetics consult follow-up note from older sibling (1/11/24)
- Laboratory A genetic testing results from older sibling (reported 12/2/2023)

April 16, 2025: Laboratory A's response to Clinical Team

After review, our genomic analysts and geneticist shared that although we are still internally suspicious that the TRMU c.652-6_652-2del variant may be pathogenic, we still formally classify it as a VUS. Please let us know if you have any additional questions.

Summary of Communication History with Laboratory B Regarding Younger Sibling

April 8, 2025: Summary of *TRMU* (NM_018006.4:c.652-6_652-2del, splicing) variant reclassification request to Laboratory B

Dear Laboratory B, I am requesting an upgrading of a VUS in the gene *TRMU*. I have attached the clinical notes for our patient (who passed away this morning) and his sister who passed away last year with exactly the same clinical presentation, down to the week of life (note plus genetic testing report from [Laboratory A]). Below is the email thread from our request to upgrade the variant at [Laboratory A] 2 years ago when this family's first child passed away of *TRMU* mitochondrial disease. Please reach out with questions or if you need additional data, as I have zero doubt this variant is pathogenic.

Attachments to email

- Medical Genetics consult follow-up note (4/7/25)
- Laboratory A genetic testing results from older sibling (reported 12/2/2023)
- Medical Genetics consult follow-up note from older sibling (1/11/24)
- *TRMU* (NM_018006.4:c.652-6_652-2del, splicing) variant reclassification request to Laboratory A for older sibling - sent via email on March 6, 2024

April 21, 2025: Laboratory B's response to Clinical Team

Thank you again for your patience as we reviewed this case. Although we agree that this is a strong variant, we are unable to upgrade the classification from a VUS at this time. We would need to observe this variant segregating in another affected relative/ in other affected families, or need a functional study to confirm the effect of the splicing variant. At this time, in silico analysis is inconclusive as to whether the variant alters gene splicing. Please let us know if we can assist further.
